# Dosimetric comparison of organs at risk in 5 different radiotherapy plans in patients with preoperatively irradiated rectal cancer

**DOI:** 10.1097/MD.0000000000024266

**Published:** 2021-01-08

**Authors:** Bekir Hakan Bakkal, Ozlem Elmas

**Affiliations:** Department of Radiation Oncology, Zonguldak Bulent Ecevit University Faculty of Medicine, Zonguldak, Turkey.

**Keywords:** conformal radiotherapy, IMRT, organs at risk, rectal carcinoma

## Abstract

**Background:**

Intensity-modulated radiotherapy (IMRT) is a widely used irradiation technique in rectal cancer patients. We aimed to compare 4 different IMRT plans with 3-dimensional conformal radiotherapy (3D-CRT) considering organs at risk (OARs) in patients with rectal carcinoma.

**Methods:**

This retrospective study included 27 rectal cancer patients who were irradiated preoperatively between January 2016 and December 2018. Five different plans (4-field 3D-CRT in 2 phases, 7-field IMRT in 2 phases, 9-field IMRT in 2 phases, 7-field simultaneous integrated boost [SIB] IMRT, and 9-field SIB IMRT) were generated for each patient. Comparison of 5 different plans according to bladder and bilateral femoral head mean doses, bladder V40, bilateral femoral head V40, and small bowel V35 values were evaluated.

**Results:**

Most of the OAR parameters significantly favored IMRT plans compared to the 3D-CRT plan. The largest difference was observed in bladder V40 values (reduction of V40 value up to 51.2% reduction) in favor of IMRT. In addition, SIB plans showed significantly better reduction in OARs than phase plans except for small bowel V35 values.

**Conclusions:**

IMRT plans reduced almost all the OARs doses compared with the 3D-CRT plan in rectal cancer patients. Furthermore, SIB plans demonstrated lower OAR doses than the phase plans. IMRT techniques, especially SIB plans, reduce OAR doses and provide safer doses for the treatment of rectal carcinoma.

## 1 Introduction

Colorectal carcinoma is the third most common malignancy of all cancers, with rectal carcinoma accounting for almost one-third of colorectal carcinomas.^[1]^ Although surgical removal of the tumor and regional lymph nodes is the main process of treatment, neoadjuvant radiotherapy (RT) with 5-fluorouracil-based chemotherapy plays a complementary role in the management of ≥cT3 or node-positive tumors by downsizing the tumor, achieving R0 resections, and reducing local recurrence rates.^[2–4]^ As local control and survival rates increase with neoadjuvant RT and mesorectal excision, more attention is required for early and late toxicities of radiation in organs at risk (OARs) in or around the target volumes, such as bladder, small bowel, and femoral heads. Many studies have shown a relationship between radiation dose and toxicities in OARs.^[5,6]^ Therefore, modern RT techniques are needed to obtain the most conformal dose distribution in target volumes with a minimum dose to OARs.

Intensity-modulated radiation therapy (IMRT) has been tried in the treatment of many pelvic malignancies and was able to reduce the dose of OARs by irradiating the target volumes with an increased conformality.^[7–9]^ Comparison of 3-dimensional conformal radiation therapy (3D-CRT) and IMRT showed better sparing of OARs, dosimetric results, and target coverage in favor of IMRT; therefore, IMRT has become the standard radiation technique in pelvis located cancers.^[10–12]^

In most of the previous studies 3D-CRT was compared with one type of IMRT plan. Unlike these studies, we aimed to compare the doses of OARs and irradiated body around the target volumes in 3D-CRT with four different IMRT plans used in rectal cancer patients who were irradiated preoperatively.

## 2 Materials and methods

### 2.1 Clinical data

Twenty-seven patients (16 males, 11 females) with rectal adenocarcinoma diagnosis who were referred to our department for preoperative irradiation between January 2016 and December 2018 were retrospectively reviewed. Inclusion criteria were patients who had pathologically proven rectal carcinoma, clinical stage T3 or N+, tumors located within 12 cm of the anal verge, and ECOG performance status 0 to 2. Our research was approved by the ethics committee of Zonguldak Bulent Ecevit University Faculty of Medicine.

Before treatment, all patients were staged by endoscopic ultrasound, thoracic/abdominopelvic computed tomography, pelvic magnetic resonance imaging, and/or positron emission tomography.

### 2.2 Simulation and target volume definition

All patients were simulated in the supine position with full bladder by drinking 1 L of water 1 hour before simulation. A computed tomography scan was performed at 3-mm intervals from the umblicus to 5 cm below the tuberosity of the ischium. The gross tumor volume (GTV) was contoured on the treatment planning system (Eclipse, Varian Medical Systems, Palo Alto, CA) taking into consideration all imaging and physical examination findings. The clinical target volume of 45 Gy (CTV 45) included a 2 cm rectum beyond the GTV, entire mesorectum, internal iliac, obturator, perirectal and presacral lymph nodes, and anal–rectal junction inferiorly. Clinical target volumes of 50 (CTV 50) and 50.4 Gy (CTV 50.4) included GTV with a 1-cm margin and involved lymph nodes. The planning target volume (PTV) was generated by 1 cm expansion of CTV 45, CTV 50, and CTV 50.4 in all directions (PTV 45, PTV 50, and PTV 50.4). Bladder, bilateral femoral heads and small bowel were contoured as OARs according to the RTOG guidelines.^[13]^ To evaluate the body dose around the target volumes, the body was contoured 5 cm below and above the PTV.

### 2.3 RT planning

Five different plans were generated for each patient:

1. 4-field 3D-CRT in 2 phases (45 Gy/25 fr + 5.4 Gy/3 fr) (PTV 45 and PTV 50.4).
2. 7-field IMRT in 2 phases (45 Gy/25 fr + 5.4 Gy/3 fr) (P7-IMRT) (PTV 45 and PTV 50.4).
3. 9-field IMRT in 2 phases (45 Gy/25 fr + 5.4 Gy/3 fr) (P9-IMRT) (PTV 45 and PTV 50.4).
4. 7-field simultaneous integrated boost (SIB) IMRT (45 Gy/25 fr - 50 Gy/25 fr) (SIB7-IMRT) (PTV 45 and PTV 50).
5. 9-field simultaneous integrated boost (SIB) IMRT (45 Gy/25 fr - 50 Gy/25 fr) (SIB9-IMRT) (PTV 45 and PTV 50).

The 4-field 3D-CRT plans were designed with gantry angle of 0, 90, 180, and 270 degree using 6 to 15 MV photons, whereas 7-field plans with 25, 75, 135, 180, 225, 280, and 325 degree, and 9-field plans with 25, 65, 100, 135, 180, 225, 260, 295, and 335 degree coplanar gantry angle using 6-MV photons. During planning and evaluation of dose–volume histograms (DVH) the dose delivered to the target volume was intended to cover at least 95% of the PTV and to achieve D2 and D98 values as recommended in ICRU-83.^[14]^ After target coverage, optimization parameters of IMRT were prioritized for dose reduction to the small bowel, bladder, and bilateral femoral heads. Limited volumes of small bowel (<200 cc) were allowed over 45 Gy if close to PTV 50/50.4, but not over 50 Gy. Various dose–volume parameters, ranging from 10 to 40 Gy, were used for small bowel volume to reduce the mean dose of small bowel to as much as possible. The V40 (percentage of volume receiving more than 40 Gy) of the bladder was kept under 35% and the maximum bladder dose was maintained at ≤54 Gy. The femoral heads were kept from receiving >45 Gy.

Bladder and bilateral femoral head doses, bladder V40, bilateral femoral head V40, and small bowel V35 values were compared in 5 different plans that were used in evaluating rectal cancer patients’ plans in our daily practice.

The treatment plans were generated using a Clinac IX (Varian Medical Systems, Palo Alto, CA) linear accelerator.

### 2.4 Statistical analysis

The data analysis was carried out using SPSS version 15.0 statistical software (SPSS, Inc., Chicago, IL). Student *t* test was used to compare dosimetric parameters between groups. A value of *P* < .05 was considered statistically significant.

## 3 Results

The median age of the 27 patients was 56 (range: 35–74) years. Patient and tumor characteristics are summarized in Table 1. DVHs were achieved for all OARs to compare the 5 plans.

**Table 1:** Patient and tumor characteristics.

Characteristic	Number	%
Sex		
Male	16	59.3
Female	11	40.7
Tumor status			
T2	8	29.6
T3	19	70.4
Nodal status			
N0	12	44.4
N+	15	55.6
Distance from anal verge, cm			
≤5	9	33.4
5.1–≤9	10	37
9.1–≤12	8	29.6

The median volumes of the small bowel, bladder, bilateral femoral heads, and body were 936 cm^3^ (range: 373–1620 cm^3^), 382 cm^3^ (range: 125–731 cm^3^), 281 cm^3^ (range: 210–369 cm^3^), and 13,918 cm^3^ (range: 9293–19,995 cm^3^), respectively.

**Figure 1:**
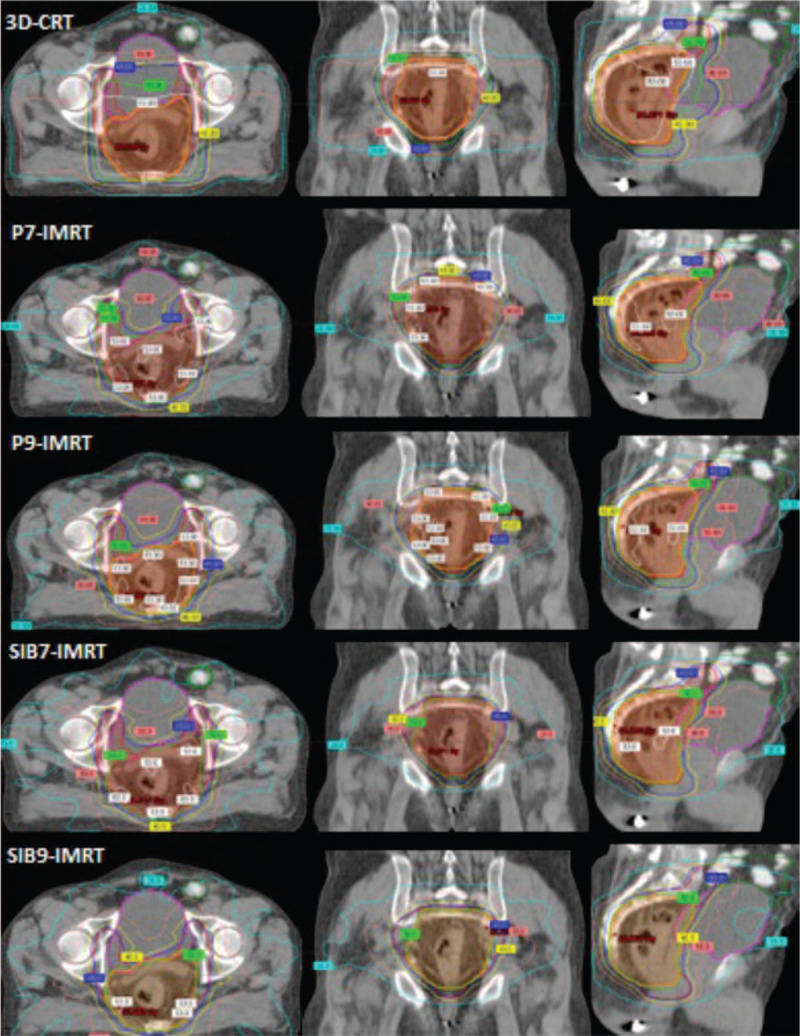
Isodose distribution of five different plans in axial, coronal and sagittal slices of the same patient. Red = PTV 45, orange = PTV 50/50.4, dark green = small bowel, pink = bladder, brown = bilateral femoral head.

All plans achieved targeted PTV coverage. Compared to 3D-CRT plans, IMRT plans showed significantly lower doses for almost all of the examined dosimetric parameters of OARs. Axial, coronal, and sagittal views of the isodose distribution of 5 different plans for 1 patient are shown in Figure 1. The outcomes of OARs are briefly explained below.

### 3.1 Small bowel

Mean small bowel V35 values in 3D-CRT, P7-IMRT, P9-IMRT, SIB7-IMRT, and SIB9-IMRT plans are mentioned in Table 2. Both phase and SIB plans significantly lowered V35 values in comparison with the 3D-CRT plan. Compared with the P7-IMRT plan, SIB7-IMRT showed similar V35 values (*P* = .45); however, the SIB9-IMRT plan had significantly lower values than P9-IMRT (*P* = .01).

**Table 2:** Dosimetric comparison of mean small bowel V35 values in 3D-CRT, P7-IMRT, P9-IMRT, SIB7-IMRT, and SIB9-IMRT plans.

Type of plan (mean small bowel V35, cc)		Change in percentage (%)	*P*
3D-CRT (138.7)	P7-IMRT (123.4)	−11	.002
	P9-IMRT (126)	−9.2	.003
	SIB7-IMRT (123.5)	−10.9	.002
	SIB9-IMRT (119.9)	−13.6	<.001
P7-IMRT (123.4)	SIB7-IMRT (123.5)	+0.1	.45
P9-IMRT (126)	SIB9-IMRT (119.9)	−4.8	.01

### 3.2 Bladder

Table 3 shows the mean bladder doses and mean bladder V40 values in 5 different plans. For the mean dose of the bladder, all IMRT plans showed significantly lower doses than the 3D-CRT plan (*P* < .001). Also, the mean bladder doses in the SIB7-IMRT and SIB9-IMRT plans were significantly lower than those in the P7-IMRT and P9-IMRT plans, respectively (*P* = .005 and *P* < .001).

**Table 3:** Dosimetric comparison of mean bladder doses and V40 values in 3D-CRT, P7-IMRT, P9-IMRT, SIB7-IMRT, and SIB9-IMRT plans.

Type of plan (mean bladder dose, Gy)		Change in percentage (%)	*P*
3D-CRT (39.8)	P7-IMRT (35)	−12.1	<.001
	P9-IMRT (34.8)	−12.6	<.001
	SIB7-IMRT (34.4)	−13.6	<.001
	SIB9-IMRT (33.5)	−15.8	<.001
P7-IMRT (35)	SIB7-IMRT (34.4)	−1.7	.005
P9-IMRT (34.8)	SIB9-IMRT (33.5)	−3.7	<.001
Type of plan (mean bladder V40 value, %)				
3D-CRT (51.7)	P7-IMRT (30.4)	−41.2	<.001
	P9-IMRT (28.8)	−44.3	<.001
	SIB7-IMRT (27.9)	−46.0	<.001
	SIB9-IMRT (25.1)	−51.2	<.001
P7-IMRT (30.4)	SIB7-IMRT (27.9)	−8.2	<.001
P9-IMRT (28.8)	SIB9-IMRT (25.1)	−12.8	<.001

In this study, IMRT showed the greatest advantage of sparing OARs in bladder V40 values. IMRT plans almost halved bladder V40 values compared with the 3D-CRT plan (*P* < .001). Furthermore, SIB-IMRT plans reduced the V40 values in comparison to 2-phase IMRT plans (*P* < .001).

### 3.3 Bilateral femoral head

With regard to femur avoidance, all of the IMRT plans provided a significant advantage in terms of bilateral femoral head sparing with respect to 3D-CRT as shown in Table 4. In addition, SIB7-IMRT and SIB9-IMRT plans lowered mean bilateral femoral head doses significantly compared with P7-IMRT and P9-IMRT plans, respectively (*P* = .002 and *P* < .001).

**Table 4:** Dosimetric comparison of mean bilateral femoral head doses and V40 values in 3D-CRT, P7-IMRT, P9-IMRT, SIB7-IMRT, and SIB9-IMRT plans.

Type of plan (mean femur dose, Gy)		Change in percentage (%)	*P*
3D-CRT (22.4)	P7-IMRT (20.1)	−10.3	.002
	P9-IMRT (18.3)	−18.8	<.001
	SIB7-IMRT (19.7)	−12.1	<.001
	SIB9-IMRT (17.9)	−20.1	<.001
P7-IMRT (20.1)	SIB7-IMRT (19.7)	−2	.002
P9-IMRT (18.3)	SIB9-IMRT (17.9)	−2.2	<.001
Type of Plan (Mean femur V40 - %)				
3D-CRT (0.9)	P7-IMRT (2.1)	+133.3	<.001
	P9-IMRT (1.5)	+66.7	.001
	SIB7-IMRT (1.6)	+77.8	.001
	SIB9-IMRT (1.2)	+33.3	.29
P7-IMRT (2.1)	SIB7-IMRT (1.6)	−23.8	<.001
P9-IMRT (1.5)	SIB9-IMRT (1.2)	−20	<.001

The dosimetric comparison for the mean bilateral femoral head V40 values in 3D-CRT, P7-IMRT, P9-IMRT, SIB7-IMRT, and SIB9-IMRT plans showed statistically significant differences (Table 4). Although all of the IMRT plans showed significantly higher doses than the 3D-CRT plan except SIB9-IMRT, the differences may not have a clinically significant difference because the maximum V40 value difference between plans (between 3D-CRT and P7-IMRT) was 1.2. Likewise, SIB7-IMRT and SIB9-IMRT showed lower V40 values than P7-IMRT and P9-IMRT, respectively, but, as the values were also close to each other and within targeted limits, the difference may not be important in daily practice.

### 3.4 Body dose

Mean body dose was quite similar in all of the plans (Table 5). Although the values were similar, P7-IMRT, SIB7-IMRT, and SIB9-IMRT plans provided significantly lower, and P9-IMRT plan significantly higher body doses in comparison to the 3D-CRT plan. In addition, comparison of P7-IMRT and P9-IMRT plans with SIB7-IMRT and SIB9-IMRT plans showed lower doses in favor of SIB plans.

**Table 5:** Dosimetric comparison of mean body dose in 3D-CRT, P7-IMRT, P9-IMRT, SIB7-IMRT, and SIB9-IMRT plans.

Type of plan (mean body dose, Gy)		Change in percentage (%)	*P*
3D-CRT (18.4)	P7-IMRT (18.2)	−1.1	<.001
	P9-IMRT (18.7)	+1.6	.02
	SIB7-IMRT (17.8)	−3.3	<.001
	SIB9-IMRT (18.2)	−1.1	.01
P7-IMRT (18.2)	SIB7-IMRT (17.8)	−2.2	<.001
P9-IMRT (18.7)	SIB9-IMRT (18.2)	−2.7	<.001

## 4 Discussion

Many acute and chronic complications caused by irradiation may occur in patients with locally advanced rectal carcinoma. Gastrointestinal, such as diarrhea, and urinary, such as dysuria, toxicities are the most common complications.^[4,15,16]^ In this study, we evaluated the dosimetric comparison of OARs in 5 different (1 3D-CRT and 4 IMRT) plans in rectal cancer patients. Many dosimetric comparisons between 3D-CRT and modern radiotherapy techniques have been published, but to our knowledge, this is the first study comparing four different IMRT plans with 3D-CRT.

A strong relationship between the doses and irradiated volumes of OARs and the severity of toxicities has been demonstrated in previous studies.^[5,17,18]^ Baglan et al^[18]^ showed a relationship between the dose of small bowel and acute diarrhea and constructed dose–volume threshold levels for acute grade 3 small bowel toxicity. They mentioned a strong association of small bowel V15 (volume receiving >15 Gy) with grade 3+ toxicity. In addition, some of the studies have shown a potent dose-volume correlation between acute diarrhea and the irradiated volume of the small bowel and constructed a predictive model for diarrhea.^[5,6]^ Therefore, all modern RT techniques aim to reduce the doses of OARs.

The effects of different treatment techniques (3D-CRT, VMAT, IMRT, among others) have been studied for many pelvic malignancies including rectal cancer.^[11,19–24]^ Almost all the studies comparing 3D-CRT and advanced RT techniques, such as IMRT, tomotherapy, and VMAT, revealed the superiority of modern techniques on OARs sparing with comparable target coverage.^[7,19,20]^ In a prospective study, 3 different RT techniques (IMRT, Rapidarc, and 3D-CRT) were compared in patients with rectal cancer and showed that IMRT and Rapidarc plans lowered V40 of the bladder, proximal femurs, colon, and small bowel significantly compared with 3D-CRT.^[23]^ Rapidarc plans also reduced Dmax of the small bowel, V40 of the right proximal femur, and V30 and Dmax of the bilateral proximal femurs compared with the IMRT plans. Furthermore, Cranmer-Sargison et al^[25]^ compared 3D-CRT and IMRT plans and showed a dose reduction in bladder, femoral head, and small bowel by 15%, 20%, and 40% with IMRT, respectively. Simson et al^[12]^ also mentioned the superiority of IMRT over 3D-CRT and showed a significant reduction in small bowel and bladder average dose, small bowel V45 and bladder V50 in their study. In our study, 4 types of IMRT plans significantly reduced almost all bladder, small bowel bilateral femoral heads and body parameters compared with the 3D-CRT plan. Only the bilateral femoral head V40 value was significantly lower in the 3D-CRT plan. Although the values were close to each other and within the targeted limits, we think this was because of the proximity of femoral heads to 40 Gy isodose. In addition, almost all SIB plans demonstrated significantly lower doses compared with phase plans. Constraints of OARs are entered once during SIB planning, but in phase planning the constraints are entered in each plan independently. This may be the reason for the better results of the SIB plans.

As modern techniques have shown reduced toxicity related to RT, VMAT, and IMRT techniques have been increasingly preferred in rectal cancer patients in recent years.^[26]^ Lee et al showed that IMRT can be used in the adjuvant treatment of rectal cancer patients with reasonable local control and tolerable RT toxicities.^[27]^ In addition, Parekh et al^[28]^ compared the toxicities of 3D-CRT and IMRT in patients with rectal carcinoma and showed a 50% reduction in acute gastrointestinal toxicity.

Some studies compared modern RT techniques with each other. Lin et al^[19]^ claimed that, although having similar target coverage, tomotherapy showed better OAR sparing, except for the small bowel, than IMRT and VMAT, and documented that supine or prone positions had no difference in sparing OARs in rectal cancer patients. Recently, Saglam et al^[29]^ evaluated a hybrid arc, a combination of double arc VMAT and forward IMRT, in locally advanced rectal cancer patients and mentioned that this hybrid technique significantly lowered most of the DVH parameters of OARs such as bladder and small bowel.

Among the studies evaluating IMRT in rectal cancer patients, a few examined the SIB technique in rectal cancer patients.^[20,30,31]^ However, all of these studies used the SIB technique as a method of IMRT treatment, not a technique to compare with 3D-CRT or phase techniques. Therefore, our study differs from other studies.

Although VMAT and IMRT have evolved the use of RT in rectal cancer, both techniques still have some limitations such as prolonged treatment duration, increased number of monitor units per fraction, and low-dose bath. We found similar but significantly lower mean body doses, but normal tissues far from the target volumes are usually exposed to low-dose radiation by modern techniques, which may trigger second malignancies induced by radiation.^[32,33]^ Although IMRT is better in OAR sparing, increased body dose and monitor unit have a risk of increased radiation-induced carcinogenesis.

Our study has some limitations, such as the small number of patients and lack of clinical outcomes because of being a dosimetric study. In addition, the total dose of 3D-CRT and phased IMRT plans were 0.4 Gy more than SIB IMRT plans, as used in daily practice. Even so, our study compared 3D-CRT and different types of IMRT plans and showed the superiority of IMRT plans over the 3D-CRT plan and SIB plans over phased plans in rectal cancer patients. We believe that the constraints of OARs can be achieved better with SIB plans than with phased plans.

## 5 Conclusion

Rectal cancer patients who received RT preoperatively have different planning options including IMRT and 3D-CRT. Our study evaluated 5 different plans and aimed to show the differences in OARs doses. Almost all of the IMRT plans significantly reduced the doses and values of OARs compared with 3D-CRT. In addition, SIB plans showed better dose reductions of OAR than phase plans. SIB planning seems to be a better option considering the doses of OARs.

## Acknowledgments

We would like to thank Mustafa Cagatay Buyukuysal for his help in statistical analysis.

## Author contributions

**Conceptualization:** Bekir Hakan Bakkal.

**Data curation:** Bekir Hakan Bakkal, Ozlem Elmas.

**Formal analysis:** Bekir Hakan Bakkal.

**Investigation:** Bekir Hakan Bakkal.

**Methodology:** Bekir Hakan Bakkal.

**Supervision:** Bekir Hakan Bakkal.

**Validation:** Bekir Hakan Bakkal, Ozlem Elmas.

**Visualization:** Bekir Hakan Bakkal.

**Writing – original draft:** Bekir Hakan Bakkal.

**Writing – review & editing:** Bekir Hakan Bakkal, Ozlem Elmas.
